# Outcome of psychogenic non-epileptic seizures following diagnosis in the epilepsy monitoring unit

**DOI:** 10.3389/fneur.2024.1363459

**Published:** 2024-02-14

**Authors:** Rachelle Abi-Nahed, Jimmy Li, Jasmine Carlier, Véronica Birca, Arline-Aude Bérubé, Dang Khoa Nguyen

**Affiliations:** ^1^Division of Neurology, Centre Hospitalier de l'Université de Montréal, Montreal, QC, Canada; ^2^Division of Neurology, Centre Hospitalier Universitaire de Sherbrooke, Sherbrooke, QC, Canada; ^3^Centre de Recherche du Centre Hospitalier Universitaire de Montréal, Montreal, QC, Canada; ^4^Département de Neurosciences, Faculté de Médecine, Université of Montréal, Montreal, QC, Canada

**Keywords:** psychogenic non-epileptic seizure, outcome, epilepsy monitoring unit (EMU), antiseizure drugs, health care utilization

## Abstract

**Objective:**

To study the outcome of patients with psychogenic non-epileptic seizures (PNES) after their diagnosis in the epilepsy monitoring unit (EMU).

**Methods:**

Patients diagnosed in our EMU with definite PNES between January 2009 and May 2023 were contacted by phone, and those who agreed to participate were asked a set of predetermined questions. Comparative analyses were carried out on several variables before and after diagnosis: number of participants with daily PNES, number of visits to the emergency department, number of participants who consulted their general practitioner or a neurologist outside of a scheduled follow-up, number of participants who took antiseizure medications (ASMs) or psychotropic drugs, and employment status.

**Results:**

Out of the 103 patients with a definite diagnosis of PNES, 61 patients (79% female) accepted to participate in our study. The median age at PNES onset was 35 years, and the median delay to diagnosis was 3 years. Almost two-thirds (62%) were receiving ASMs and 40% psychotropic drugs. The mean stay at the EMU was 5 days. PNES diagnosis was explained to almost all patients (97%) by the end of their EMU stay and was well-accepted by most (89%). When contacted, 46% of participants no longer had PNES; 32% mentioned that their PNES had ceased immediately upon communication of the diagnosis. The median follow-up duration was 51 months. Fewer patients had daily seizures after the diagnosis (18 vs. 38%; *p* < 0.0455). Similarly, the median number of emergency department visits was significantly lower (0 vs. 2; *p* < 0.001). Only 17 patients consulted their general practitioner (vs. 40, *p* < 0.001) and 20 a neurologist (vs. 55, *p* < 0.001) after a PNES attack outside of a scheduled follow-up. The use of ASMs was also significantly reduced from 70 to 33% (*p* < 0.01), with only one still taking an ASM for its antiseizure properties. Significantly more participants were working at last follow-up than at PNES diagnosis (49 vs. 25%; *p* < 0.001).

**Conclusion:**

Our study revealed a relatively favorable long-term outcome of definite PNES diagnosed in the EMU that translated in significant reductions in PNES frequency, health care utilization and ASM use, as well as a significant increase in employment rate.

## Introduction

Psychogenic non-epileptic seizures (PNES) are heterogeneous paroxysmal attacks that resemble epileptic seizures but are not caused by abnormal epileptiform discharges. Previously referred to as “conversion disorder,” PNES are currently designated as a “Functional Neurological Disorder” (FND) in the latest DSM-V-TR (1). Recent estimates place the prevalence of PNES at 50/100,000 (2). While the underlying pathophysiology remains uncertain, most believe that PNES is far more complex than a simple non-structural epilepsy mimic or a straightforward somatic manifestation of an inner distress. An integrative cognitive model has been developed to dissect the causal processes (3). Another biopsychosocial-based model emphasizes the place of predisposing factors such as female gender, previous sexual abuse or neglect in childhood, and comorbid psychiatric conditions (4). More recently, an innovative approach based on predictive brain processing has emerged in the understanding of FND (5).

The International League Against Epilepsy (ILAE) Non-epileptic Seizures Task Force issued in 2011 and 2013 recommendations on the management of these patients (6, 7). These recommendations include diagnosis by video-EEG monitoring, adequate communication of the diagnosis to the patient and his relatives, a gradual withdrawal of antiseizure medications (ASMs) in patients with a diagnosis of PNES without concomitant epilepsy, the prescription of psychotropic drugs for comorbid psychopathologies, and long-term follow-up of patients.

It has previously been reported that patients with PNES have a good outcome in the short-term (6). However, the long-term fate of these patients is still unclear. Some studies have found that beyond 1 year after diagnosis, < 40% of patients have complete control of their PNES (8–12). On the contrary, other studies have reported more encouraging results: beyond 1 year after diagnosis, 63% of patients reported having had complete control of their PNES (during the last 3 months), and 76.1% of patients reported that their last episode was more than a month ago (13, 14). Most of these studies do not distinguish between patients with PNES only and those with concomitant epilepsy. Sadan et al. (15) suggested that cessation of long-term PNES in patients with concurrent epilepsy may be more likely than in patients with PNES alone, whereas Meierkord et al. (16) suggested otherwise. It is difficult to compare these two groups (PNES only vs. PNES with concomitant epilepsy) because studies indicate that a significant proportion of patients and their caregivers cannot discriminate PNES from epileptic seizures 1 year after diagnosis (17). Moreover, although control of PNES is important and is the main measure of their outcome in studies, it is not a representative measure of medical or psychosocial outcome (8, 10). Thus, Durrant et al. (8) suggested that studies should use a wider variety of measures including economic status, overall level of functioning, and other indicators of quality of life. For example, some studies have examined, in addition to the number of PNES, measures such as economic status, health care utilization, and prescription of drugs. Results have varied across studies.

This is to our knowledge the first study of PNES prognosis in a Canadian population. We combined several of the aforementioned outcome measurements (PNES frequency, use of ASMs, utilization of healthcare resources, and socioeconomic status) to paint a broad picture of the clinical outcomes of patients diagnosed with PNES in our epilepsy monitoring unit (EMU).

## Methods

### Participants

This study was approved by the CHUM's Ethics Committee. Using our EMU database, all patients diagnosed with definite PNES between January 2009 and May 2023 were identified. A definite diagnosis of PNES was made by combining the history from patients/witnesses and video-EEG in order to ensure gold standard diagnosis with highest levels of certainty and reliability. Once the ILAE diagnostic criteria were published in 2011 (18), case ascertainment was made using these criteria; however, even before these criteria were published, diagnosis was made similarly (19). Patients with only probable PNES or other entities of FND were excluded. Patients exhibiting both epileptic seizures and PNES were also excluded considering that a significant proportion of patients and their caregivers cannot discriminate PNES from epileptic seizures (17).

Along with a consent form and a return envelope, an introductory letter was sent to all identified patients to summarize the objectives of the study and inform them that someone from the research team (RAN) would phone them during the month of July 2023. Patients who agreed to participate underwent a phone interview with a set of predetermined questions. Consent to consult their medical chart and their Quebec Health Booklet (an online service containing the list of their medications, lab results, and medical imaging reports) was also obtained.

### Data collection

Collected information included demographical data (gender, age), information relating to PNES (semiology, frequency, co-morbidities, and prescribed medications) and outcome data (PNES frequency, number of visits to the emergency department, general practitioners or neurologists, use of antidepressants, antipsychotics, or ASMs, and ability to continue working or to return to work after a leave). This data was obtained from every patient during the phone interview and compared to the information written in the medical record when available; in case of discrepancies (which were seldom), the answers provided by patients during the phone interview were retained.

### Data analysis

All statistical analyses were performed using R version 4.3.1. Descriptive analyses were first carried out on all collected data for all participants (demographic characteristics and comorbidities, diagnosis-related data, PNES semiology, burden, and prognostic outcomes). Continuous data are presented as medians (IQR, interquartile range), and binary/categorical data are presented as count (proportion).

Comparative analyses were carried out on relevant PNES burden variables: number of participants with daily PNES, number of visits to the emergency department, number of participants who consulted their general practitioners or a neurologist, number of participants who took antidepressants, antipsychotics, or ASMs, and the number of participants who partook in psychotherapy sessions. These variables were compared pre- and post-diagnosis of PNES. Continuous variables were compared using paired Mann Whitney *U*-tests due to the non-parametric distribution of these variables. Binary variables were compared using McNemar's tests. Paired tests were used since comparisons were made for the same participants across time. Significance level was set at 0.05. Missing data were treated with pairwise deletion.

## Results

### Participants

From our EMU database, we identified 360 patients with a diagnosis of non-epileptic seizures between January 2009 and May 2023. During the pre-selection phase, 257 patients (71%) were excluded for various reasons: physiological (not psychogenic) non-epileptic seizures, PNES with concomitant epilepsy, probable (not definite) PNES, or deceased. Out of the 103 patients with a definite diagnosis of PNES, 29 could not be reached, and 13 declined to participate, leaving 61 patients with a definite diagnosis of PNES willing to engage in our study (response rate = 59.2%). The participant selection process is detailed in Supplementary Figure 1. These 61 participants were diagnosed over the years as such: 3 in 2011, 1 in 2012, 4 in 2013, 2 in 2014, 3 in 2015, 7 in 2016, 3 in 2017, 7 in 2018, 7 in 2019, 4 in 2020, 10 in 2021, 8 in 2022, and 2 in 2023 (until May 2023). This resulted in a follow-up range of 2 months to almost 12 years.

#### Before PNES diagnosis

Table 1 details the general characteristics of PNES patients upon admission to the EMU. Most participants were of female gender (79%). Forty-three (70%) patients had a job of whom 28 took a temporary leave of absence; 14 were still students. The median age at onset of PNES was 35 years (18–44) and the median delay between PNES onset and diagnosis was 3 years (1–7). Many (61%) reported at least one psychiatric comorbidity (46% anxiety disorders, 39% depressive disorders, and 16% personality disorders). A history of sexual abuse and physical abuse was reported by 21 (34%) and 4 (7%) patients, respectively. Additional information on psychiatric comorbidities and lifestyle habits are provided in Supplementary Table 2.

**Table 1:** General characteristics of participants before and at PNES diagnosis.

	**n (%)**	**Median (IQR)**	**N**
**Demographics**			
**Female sex**	48 (79)		61
**Age at onset of PNES, years**		35 (18–44)	61
**In a relationship (vs. single/widowed)**	42 (69)		61
**Employment status at the time of the diagnosis**			61
Employed and working	15 (25)		
On temporary leave from work	28 (46)		
Student	14 (23)		
Unemployed	3 (5)		
Retired	1 (2)		
**Highest level of education**			61
Primary school	4 (7)		
High school	16 (26)		
CEGEP	21 (34)		
University	15 (25)		
Special education	5 (8)		
**Comorbidities**			
**Psychiatric disorders** ^**a**^	42 (69)		61
Anxiety disorders	28 (46)		
Depressive disorders	24 (39)		
Personality disorder	10 (16)		
**Functional symptoms**	34 (56)		61
Chronic pain	19 (31)		
Chronic headache	17 (28)		
Irritable bowel syndrome	18 (30)		
**History of sexual abuse**	21 (34)		61
**History of physical abuse**	4 (7)		61
**Diagnosis-related**			
**Delay between PNES onset and diagnosis, years**		3 (1–7)	61
**Time spent at EMU, days**		5 (3–8)	61
**Number of PNES attacks recorded in EMU**		3 (1–5)	61
**PNES diagnosis announced at EMU**	59 (97)		61
**FND program referral made at EMU discharge**	18 (48)		37

CEGEP, College of General and Professional Teaching; CEGEP is a publicly funded college that is exclusive to the province of Quebec's education system. EMU, epilepsy monitoring unit; IQR, interquartile range; n, count; N, number of complete cases; PNES, psychogenic non-epileptic seizures.

^a^Details regarding exact psychiatric comorbidities and lifestyle habits can be found in Supplementary Table 2.

**Figure 1:**
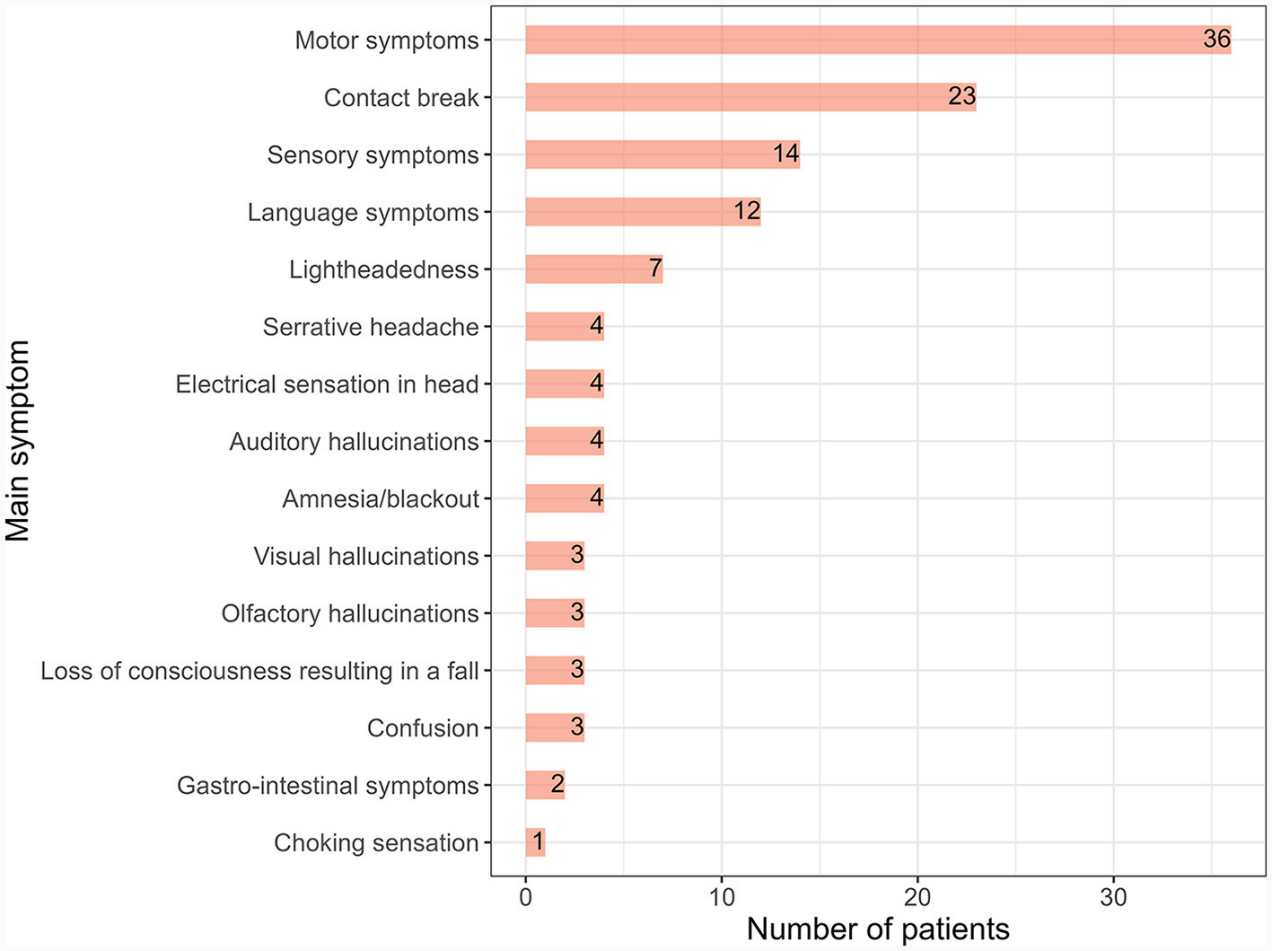
PNES semiology before diagnosis. PNES, psychogenic non-epileptic seizure. Each participant could have more than one clinical feature. In total, 36/61 (59%) participants had more than one clinical feature.

**Figure 2:**
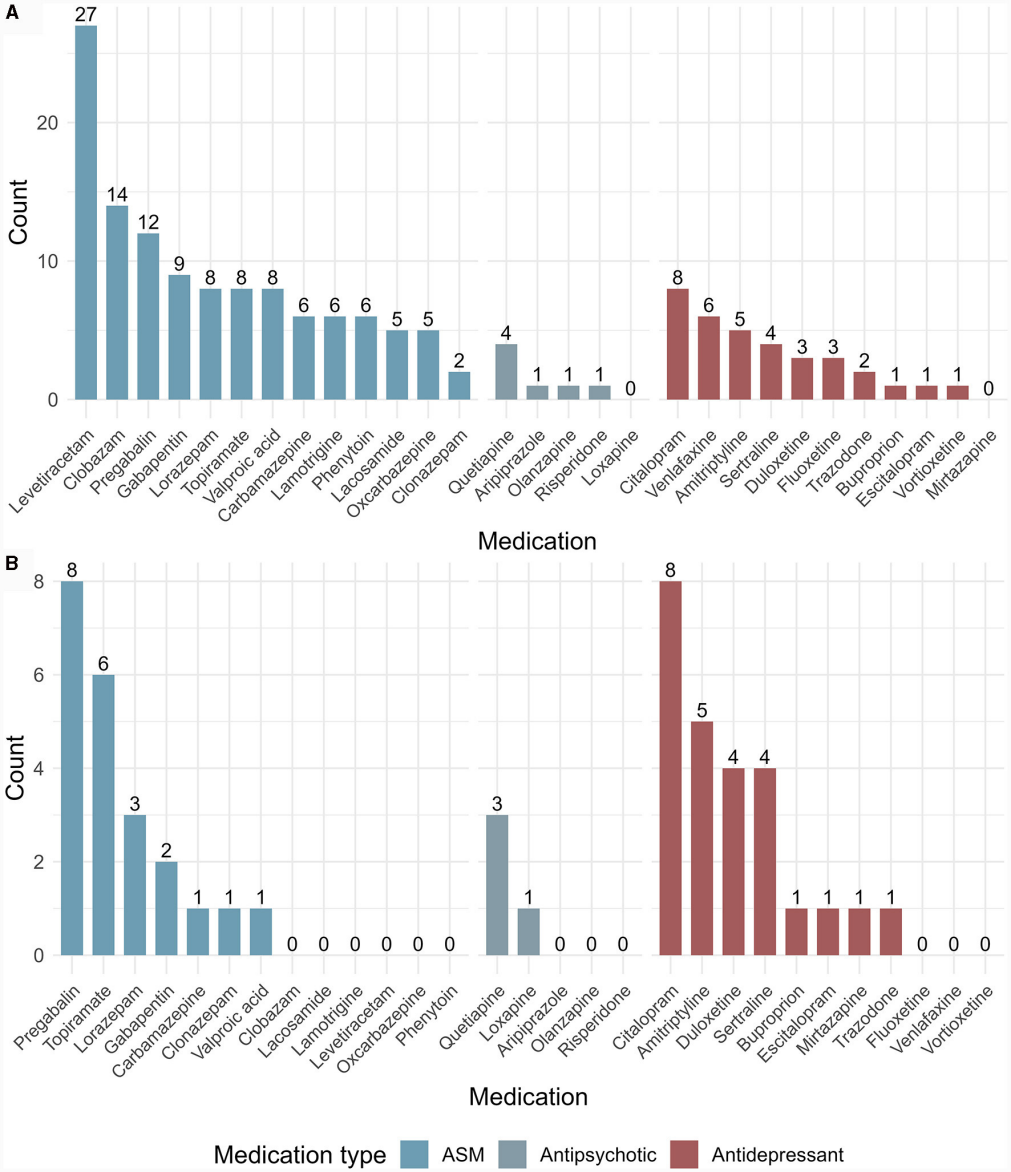
Antiseizure medications, antipsychotics, and antidepressants taken by participants before **(A)** and after **(B)** PNES diagnosis. ASM, antiseizure medication; PNES, psychogenic non-epileptic seizure. Each participant could take more than one medication.

The majority (59%) of patients had PNES featuring overt motor symptoms, and most (59%) had more than one clinical manifestation (Figure 1). Thirty-eight percent of patients reported a maximum PNES burden—throughout their PNES course—of daily attacks and 38% of weekly attacks (Table 2). Before PNES diagnosis, 62% were receiving antiseizure therapy, including 18% receiving more than one ASM. Forty percent of patients were taking psychotropic drugs, including 15% taking more than one antipsychotic or antidepressant. Figure 2A depicts which ASM, antipsychotics, and antidepressants were used by participants before their PNES diagnosis.

**Table 2:** Burden of PNES on participants before and after diagnosis.

	**Before PNES diagnosis**			**After PNES diagnosis**			
	**Median (IQR)**	**n (%)**	**N**	**Median (IQR)**	**n (%)**	**N**	**p-value**
Had daily PNES during the course of their PNES		23 (38)	61		6 (18)	33	**0.0455**
# of ED visits for PNES	2 (1–4.25)		60	0 (0–0)		59	**< 0.001**
Consulted a general practitioner for PNES outside a scheduled follow up		40 (66)	61		17 (28)	61	**< 0.001**
Consulted a neurologist for PNES outside a scheduled follow up		55 (90)	61		20 (33)	61	**< 0.001**
Took antipsychotics or antidepressants		31 (51)	61		25 (41)	61	0.302
Took ASMs		43 (70)	61		20 (33)	61	**< 0.001**
Had psychotherapy sessions		21 (34)	61		25 (41)	61	0.480
Employed and working		15 (25)	61		30 (49)	61	**< 0.001**

ASM, antiseizure medication; ED, emergency department; IQR, interquartile range; n, count; N, number of complete cases; PNES, psychogenic non-epileptic seizures.

Continuous variables were compared using paired Mann-Whitney U-tests, and binary variables were compared using McNemar's tests. The significance level was set at 0.05. Statistically significant results are shown in bold.

The majority (70%) reported having gone to the emergency department or to see a healthcare professional (general practitioner or neurologist) within 3 months of their symptom onset. The median number of times participants visited the emergency department before EMU admission was 2 (1–4.25).

#### Upon PNES diagnosis

The median stay at the EMU was 5 days (3–8). A median of 3 (1–5) PNES attacks per patient were recorded in the EMU. PNES diagnosis was explained to almost all patients (97%) by the end of their EMU stay and was well-accepted by most (89%). The EMU report was sent to their general practitioner and to every specialist involved in the patient's care. Thirty-five patients (57%) had a scheduled follow-up with an epileptologist from our institution a few months after diagnosis at the EMU, mostly after 2018 (27 out of 35; 77%).

Referral and care or follow-up at our FND clinic (established in 2018) was done for 17 of the 36 patients (47%) diagnosed after 2018. The remaining 19 patients were not referred for the following reasons: 3 had spontaneous PNES resolution upon diagnosis, 10 already had significantly fewer PNES upon early follow-up, and 6 already had a psychiatrist/psychotherapist following them.

#### After PNES diagnosis

Table 2 presents the univariate analyses of PNES burden variables performed pre- and post-diagnosis of PNES.

### Occurrence of PNES

**Figure 3:**
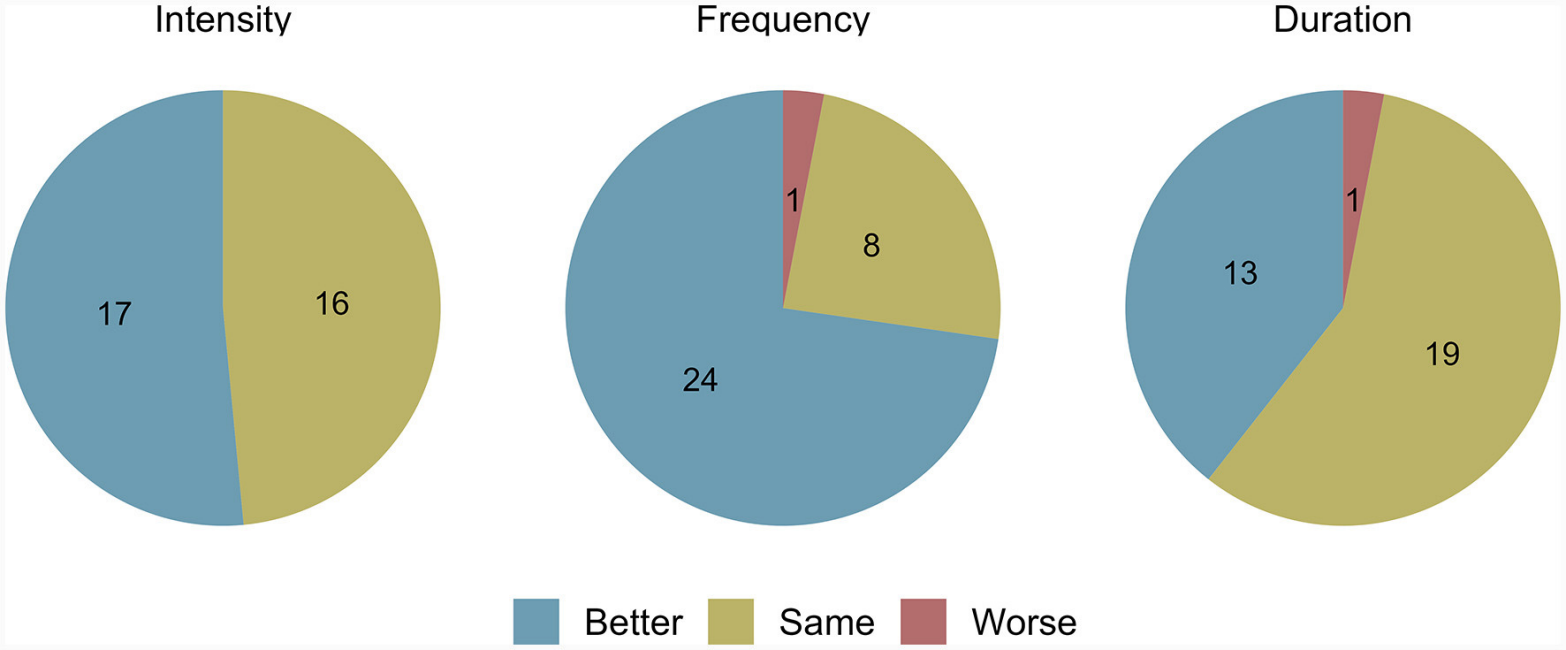
Prognostic outcomes in participants who still had PNES at data collection. PNES, psychogenic non-epileptic seizures. The intensity, frequency, and duration of PNES in participants who still had such episodes at data collection are depicted here. Only 33 participants had usable data, as the other 28 were free of PNES.

PNES freedom was defined as a complete absence of PNES at time of follow-up. At the time of data collection by phone, 28 out of 61 (46%) participants had achieved PNES freedom, 9 of whom (32%) mentioned that episodes stopped immediately upon communication of the diagnosis at the end of their EMU stay. Overall, the median time required to achieve PNES freedom after diagnosis was 3.5 months (0–15). Also, significantly fewer patients had daily seizures (18 vs. 38%; *p* < 0.0455). The median follow-up duration was 51 months (23–86); 93% of participants had a follow-up of more than a year. Among the 33 participants who still had PNES, 73% felt that they were fewer, 52% less intense, and 39% shorter (Figure 3). Supplementary Table 3 lists the factors participants believed led to PNES freedom. These were mainly lifestyle modifications to prioritize mental health, psychotherapy, better stress management, and understanding the diagnosis.

### Use of ASMs and psychotropics

The use of ASMs was significantly reduced from 70 to 33% (*p* < 0.01). Among the 20 patients still taking an ASM after PNES diagnosis, only one was doing so to treat “seizures;” the remaining patients were taking ASM(s) to treat conditions other than “seizures” (notably pain, anxiety, insomnia, and bipolar disorder). There was no significant change in antidepressant, antipsychotic, or psychotherapy use after diagnosis. Figure 2B depicts which ASMs, antipsychotics, and antidepressants were used by participants after their PNES diagnosis.

### Health care utilization

The median number of emergency department visits was significantly lower after diagnosis (0 vs. 2; *p* < 0.001). Only 17 patients consulted their general practitioner (vs. 40 pre-diagnosis, *p* < 0.001), and only 20 consulted a neurologist (vs. 55, *p* < 0.001) outside of a scheduled follow-up for a PNES attack.

### Socioeconomic status

Supplementary Table 4 depicts the participants' pre- and post-PNES-diagnosis employment status.

Among the 14 students, nine were still pursuing their studies, and five had started working. As for the 28 patients on temporary leave from work, only 10 (32%) went back to work, and 2 retired. Out of the 16 people who were not able to go back to work, six were PNES-free. Two of these six subjects were already on work leave before PNES occurrence, three did not return to work for psychiatric reasons and one was close to retirement. In total, significantly more participants were working at follow-up than at PNES diagnosis (49 vs. 25%; *p* < 0.001).

## Discussion

In this study, we sought to assess the long-term outcome of a cohort of 61 patients diagnosed with definite PNES in our EMU. In line with previous cohorts, our patients were predominantly females (79%) with a median age of 35 years at PNES onset (20–22). A third of the subjects reported a history of sexual abuse and 69% had at least one psychiatric comorbidity (46% anxiety, 39% depressive, and 16% personality disorders). Half of patients demonstrated co-occurrence of PNES with another functional disorder (such as chronic pain and irritable bowel syndrome), confirming the close overlap among FND and other functional symptomatologies (1, 23, 24). As expected, the impact of PNES is significant: more than half of patients with a job stopped working; two thirds were receiving antiseizure therapy; 70% patients reported consulting a healthcare professional within 3 months of their symptom onset; and the median number of visits to the emergency department before PNES diagnosis was 2.

Fortunately, close to half of our cohort showed complete remission and only 18% of patients reported daily PNES after diagnosis (vs. 38% before). These results are on the higher end of PNES remission range reported in previous studies. Throughout the years, different outcomes have been reported in the literature. Gelauff et al. (25) collected data on PNES outcome from two previous reviews (8, 26) concerning 25 studies from 1990 until 2016, in different countries with different cohort sizes (15–188 patients), and different follow-up periods (3 months−8 years). A complete PNES remission score equal or < 40% was found in 16 out of these 25 studies, with an average of 33% [ranging from 7% as reported by Jones et al. (27) to 58% as reported by Buchanan and Snars (28)]. More recent studies have also reported divergent outcomes; for example, Asadi-Pooya et al. (29) found a 54.7% PNES remission rate whereas Massot-Tarrus et al. (30) only 26.8%. Several reasons underlie these heterogeneous findings: cohorts are very disparate in size and characteristics, follow-up durations vary from 2 weeks to 14 years (7), and PNES freedom is not uniformly defined. However, although a low rate of complete recovery has been frequently reported, half of the studies interestingly describe a significant reduction in the number of daily seizures after diagnosis (including a few studies with follow-up periods beyond 4 years).

We also found a significant reduction in ASM use after PNES diagnosis from 70 to 33% (*p* < 0.01). These numbers are in line with previously published rates of approximately two-thirds of patients receiving ASM prior to PNES diagnosis (28, 31) and a persistence of around 25–40.7% of patients still taking ASM at 4 years after the diagnosis (11, 31, 32). In our cohort, only one out of the 20 patients still taking ASMs did so for its antiseizure properties as the others were taking them to treat comorbidities such as pain and anxiety. To our knowledge, the majority of studies do not specifically explore, at an individual level, the reasons why patients continued to take ASMs after PNES diagnosis. Only one study published in 2000 did so (33) with the authors reporting a considerable amount of patients able to discontinue ASM at follow-up; 3/57 refused to stop the ASMs and 4 continued to take ASMs for pain or mood stabilization. It has been reported that patients with ongoing PNES are more likely to use ASMs (32); Reuber et al. (11) showed a correlation between persistent PNES and the use of ASMs. Several authors highlight the potential role of poor coordination among patients, medical referrers, and the epilepsy center in the persistence of ASM prescriptions (25, 31, 33), showcasing the importance of informing local medical referrers that their patients have PNES and thus no longer require ASMs.

We also found a significant decrease in visits to the emergency department, general practitioners, and neurologists after PNES diagnosis which theoretically should translate into a reduction in healthcare costs. A few studies, mainly in the United States and recently in Australia, have scrutinized health care utilization cost caused by PNES (34–38). They all showcased the considerable burden that PNES has on the health care system. Anderson et al. (39) found a significant reduction of 92% in the total average contacts (included emergency department visits, hospital admissions, outpatient clinic appointments, and brain imaging) in 24 patients diagnosed with PNES alone. Conversely, Ramamurthy et al. (40) found that 23% visited the emergency department a month after PNES diagnosis, and Salinsky et al. (41) found no overall improvement in health care utilization during the 3 years following PNES diagnosis compared to 3 years before diagnosis. Another promising finding was the significant increase in employment rate; the number of patients working actually doubled after the PNES diagnosis (49 vs. 25%), and only 16 out of 43 patients (37%) who had a job were unable to return to work; this rate is lower than what has been reported previously showing a range of 43–89% (25) of patients with PNES unable to regain their professional lives.

Overall, our cohort seemed to have a good outcome, somewhat better than what had been described in several past studies. We surmise that these positive results are partly the results of the work done by the scientific community over the last decade on PNES, starting with recommendations made by the ILAE PNES Task Force (42, 43) and the FND Society (44). First, PNES diagnosis was confirmed in the EMU using video-EEG (the gold standard). Our cohort demonstrated a high rate of diagnostic acceptance (89%), which may have contributed to a certain degree to PNES outcome improvement. Second, the diagnosis was clearly announced at the end of the EMU stay in 97% of cases. Many studies agree wholeheartedly that a clearly and honestly communicated diagnosis leads to a better outcome (33–35). Third, the EMU video-EEG monitoring report was shared with healthcare professionals involved with the patient's care, limiting the chances that ASMs would be restarted. Similarly, the fact that the majority of patients had a scheduled follow-up with an epileptologist from our institution, especially after 2018, could have contributed to the low number of patients taking an ASM for “seizure” therapy purposes. Although it is still early for any hard conclusions, the creation in 2018 of a dedicated FND clinic with a Bayesian approach (45) (which provides multidisciplinary neuropsychiatric consultations, physiotherapy, and occupational therapy specialized in FND) could also have partly contributed to the overall good outcome.

This study had several strengths, including a diverse sample, a good response rate, a long follow up period and the use of several parameters to document the outcome. Its limitations include the modest sample size (similar to prior studies). Another limitation was generalizability due to recruitment issues; patients who agreed to participate might have had a better prognosis. Thus, our results might overestimate the improvement in outcome. Moreover, the retrospective nature of this study makes it susceptible to recall bias. We checked all available data within each patient's medical chart and their Quebec Health Records to reduce recall bias, particularly for patients diagnosed with PNES more than 10 years ago. However, when patients were not followed or no longer followed at our institution, we had to rely on patient recollection which may not necessarily be accurate. Finally, all our patients were recruited from a tertiary epilepsy center and are thus not necessarily representative of the population of PNES in non-academic centers.

## Conclusion

Our study reveals a relatively favorable long-term outcome of definite PNES diagnosed in the EMU that translates in PNES freedom or significant reduction in PNES frequency, ASM discontinuation, and a significant reduction in health care utilization. Future studies are needed to assess how dedicated/specialized FND clinics can impact the outcome of PNES patients.

## Data availability statement

The original contributions presented in the study are included in the article/Supplementary material, further inquiries can be directed to the corresponding authors.

## Ethics statement

The studies involving humans were approved by Centre Hospitalier de l'Université de Montréal (CHUM)'s Ethics Committee. The studies were conducted in accordance with the local legislation and institutional requirements. The participants provided their written informed consent to participate in this study.

## Author contributions

RA-N: Conceptualization, Data curation, Formal analysis, Investigation, Methodology, Project administration, Software, Supervision, Validation, Writing – original draft, Writing – review & editing. JL: Conceptualization, Methodology, Software, Writing – review & editing. JC: Conceptualization, Writing – review & editing. VB: Writing – review & editing. A-AB: Writing – review & editing. DN: Supervision, Validation, Writing – review & editing.

## References

[1] American Psychiatric Association. DSM-5: Diagnostic and Statistical Manual of Mental Disorders. 5th ed. Washington, DC: American Psychiatric Association (2013).

[2] Kanemoto K LaFrance WC Duncan R Gigineishvili D Park SP Tadokoro Y. PNES around the world: where we are now and how we can close the diagnosis and treatment gaps—an ILAE PNES Task Force report. Epilepsia Open. (2017) 2:307–16. 10.1002/epi4.12060 29588959 PMC5862115

[3] Brown RJ Reuber M. Psychological and psychiatric aspects of psychogenic non-epileptic seizures (PNES): a systematic review. Clin Psychol Rev. (2016) 45:157–82. 10.1016/j.cpr.2016.01.003 27084446

[4] Reuber M Howlett S Khan A Grünewald RA. Non-epileptic seizures and other functional neurological symptoms: predisposing, precipitating, and perpetuating factors. Psychosomatics. (2007) 48:230–8. 10.1176/appi.psy.48.3.230 17478592

[5] Jungilligens J Paredes-Echeverri S Popkirov S Barrett LF Perez DL. A new science of emotion: implications for functional neurological disorder. Brain. (2022) 145:2648–63. 10.1093/brain/awac204 35653495 PMC9905015

[6] Lafrance WC Reuber M Goldstein LH. Management of psychogenic nonepileptic seizures. Epilepsia. (2013) 54:53–67. 10.1111/epi.12106 23458467

[7] Kerr MP Mensah S Besag F de Toffol B Ettinger A Kanemoto K. International consensus clinical practice statements for the treatment of neuropsychiatric conditions associated with epilepsy. Epilepsia. (2011) 52:2133–8. 10.1111/j.1528-1167.2011.03276.x 21955156

[8] Durrant J Rickards H Cavanna AE. Prognosis and outcome predictors in psychogenic nonepileptic seizures. Epilepsy Res Treat. (2011) 2011:1–7. 10.1155/2011/274736 22937230 PMC3428611

[9] Lancman ME Brotherton TA Asconapé JJ Kiffin Penry J. Psychogenic seizures in adults: a longitudinal analysis. Seizure. (1993) 2:281–6. 10.1016/S1059-1311(05)80141-4 8162396

[10] Reuber M Mitchell AJ Howlett S Elger CE. Measuring outcome in psychogenic nonepileptic seizures : how relevant is seizure remission? Epilepsia. (2005) 46:1788–95. 10.1111/j.1528-1167.2005.00280.x 16302859

[11] Reuber M Pukrop R Bauer J Helmstaedter C Tessendorf N Erich Elger C. Outcome in psychogenic nonepileptic seizures: 1 to 10-year follow-up in 164 patients. Ann Neurol. (2003) 53:305–11. 10.1002/ana.3000 12601698

[12] Walther K Volbers B Erdmann L Onugoren MD Gollwitzer S Kasper BS. Psychological long-term outcome in patients with psychogenic nonepileptic seizures. Epilepsia. (2019) 60:669–78. 10.1111/epi.14682 30838655

[13] Duncan R Graham CD Oto M. Outcome at 5-10years in psychogenic nonepileptic seizures: what patients report vs. what family doctors report. Epilepsy Behav. (2014) 37:71–4. 10.1016/j.yebeh.2014.06.011 25010317

[14] Gambini O Demartini B Chiesa V Turner K Barbieri V Canevini MP. Long-term outcome of psychogenic nonepileptic seizures: the role of induction by suggestion. Epilepsy Behav. (2014) 41:140–3. 10.1016/j.yebeh.2014.09.076 25461206

[15] Sadan O Neufeld MY Parmet Y Rozenberg A Kipervasser S. Psychogenic seizures: long-term outcome in patients with and without epilepsy. Acta Neurol Scand. (2016) 133:145–51. 10.1111/ane.12458 26177156

[16] Meierkord H Will B Fish D Shorvon S. The clinical features and prognosis of pseudoseizures diagnosed using video-EEG telemetry. Neurology. (1991) 41:1643–6. 10.1212/WNL.41.10.1643 1922808

[17] Gordon PC Valiengo LDCL Proença ICGF Kurucgant D Jorge CL Castro LH. Comorbid epilepsy and psychogenic non-epileptic seizures: how well do patients and caregivers distinguish between the two. Seizure. (2014) 23:537–41. 10.1016/j.seizure.2014.04.002 24795150

[18] Lafrance WC Baker GA Duncan R Goldstein LH Reuber M. Minimum requirements for the diagnosis of psychogenic nonepileptic seizures: a staged approach: a report from the International League Against Epilepsy Nonepileptic Seizures Task Force. Epilepsia. (2013) 54:2005–18. 10.1111/epi.12356 24111933

[19] Gasparini S Beghi E Ferlazzo E Beghi M Belcastro V Biermann KP. Management of psychogenic non-epileptic seizures: a multidisciplinary approach. Eur J Neurol. (2019) 26:205–15. 10.1111/ene.13818 30300463

[20] Carson A Lehn A. Epidemiology. Handb Clin Neurol. (2016) 139:47–60. 10.1016/B978-0-12-801772-2.00005-9 27719864

[21] Hingray C Biberon J El-Hage W De Toffol B. Psychogenic non-epileptic seizures (PNES). Rev Neurol. (2016) 172:263–9. 10.1016/j.neurol.2015.12.011 27117433

[22] Duncan R Oto M Martin E. Diagnostic delay in psychogenic nonepileptic seizures. Neurology. (2002) 58:493–5. 10.1212/WNL.58.3.493 11839862

[23] Nicholson TR Carson A Edwards MJ Goldstein LH Hallett M Mildon B. Outcome measures for functional neurological disorder: a review of the theoretical complexities. J Neuropsychiatry Clin Neurosci. (2020) 32:33–42. 10.1176/appi.neuropsych.19060128 31865871

[24] Stone J Carson A Duncan R Roberts R Coleman R Warlow C. Which neurological diseases are most likely to be associated with “symptoms unexplained by organic disease.” J Neurol. (2012) 259:33–8. 10.1007/s00415-011-6111-0 21674198

[25] Gelauff J Stone J. Prognosis of functional neurologic disorders. Handb Clin Neurol. (2016) 139:523–41. 10.1016/B978-0-12-801772-2.00043-6 27719869

[26] Gelauff J Stone J Edwards M Carson A. The prognosis of functional (psychogenic) motor symptoms: a systematic review. J Neurol Neurosurg Psychiatry. (2014) 85:220–6. 10.1136/jnnp-2013-305321 24029543

[27] Jones SG O'Brien TJ Adams SJ Mocellin R Kilpatrick CJ Yerra R. Clinical characteristics and outcome in patients with psychogenic nonepileptic seizures. Psychosom Med. (2010) 72:487–97. 10.1097/PSY.0b013e3181d96550 20368472

[28] Buchanan N Snars J. Pseudoseizures (non epileptic attack disorder)—clinical management and outcome in 50 patients. Seizure. (1993) 2:141–6. 10.1016/S1059-1311(05)80119-0 8167966

[29] Asadi-Pooya AA Ziyaee F. Outcome of patients with psychogenic nonepileptic seizures with limited resources: a longitudinal study. Seizure. (2018) 59:1–4. 10.1016/j.seizure.2018.04.017 29709720

[30] Massot-Tarrús A Joe Yu Y AlKhateeb M Mirsattari SM. Predicting outcome of patients with psychogenic nonepileptic seizures after diagnosis in an epilepsy monitoring unit. Epilepsy Behav. (2021) 120:108004. 10.1016/j.yebeh.2021.108004 33984657

[31] Reuber M Elger CE. Psychogenic nonepileptic seizures: review and update. Epilepsy Behav. (2003) 4:205–16. 10.1016/S1525-5050(03)00104-5 12791321

[32] Volbers B Walther K Kurzbuch K Erdmann L Gollwitzer S Lang JD. Psychogenic nonepileptic seizures: clinical characteristics and outcome. Brain Behav. (2022) 12:1–11. 10.1002/brb3.2567 35413160 PMC9120718

[33] Selwa LM Geyer J Nikakhtar N Brown MB Schuh LA Drury I. Nonepileptic seizure outcome varies by type of spell and duration of illness. Epilepsia. (2000) 41:1330–4. 10.1111/j.1528-1157.2000.tb04613.x 11051130

[34] Ahmedani BK Osborne J Nerenz DR Haque S Pietrantoni L Mahone D. Diagnosis, costs, and utilization for psychogenic non-epileptic seizures in a US health care setting. Psychosomatics. (2013) 54:28–34. 10.1016/j.psym.2012.08.005 23194931

[35] Binder LM Salinsky MC. Psychogenic nonepileptic seizures. Neuropsychol Rev. (2007) 17:405–12. 10.1007/s11065-007-9047-5 18041588

[36] Magee JA Burke T Delanty N Pender N Fortune GM. The economic cost of nonepileptic attack disorder in Ireland. Epilepsy Behav. (2014) 33:45–8. 10.1016/j.yebeh.2014.02.010 24632352

[37] Martin RC Gilliam FG Kilgore M Faught E Kuzniecky R. Improved health care resource utilization following video-EEG-confirmed diagnosis of nonepileptic psychogenic seizures. Seizure. (1998) 7:385–90. 10.1016/S1059-1311(05)80007-X 9808114

[38] Seneviratne U Low ZM Low ZX Hehir A Paramaswaran S Foong M. Medical health care utilization cost of patients presenting with psychogenic nonepileptic seizures. Epilepsia. (2019) 60:349–57. 10.1111/epi.14625 30577087

[39] Anderson J Hill J Alford M Oto M Russell A Razvi S. Healthcare resource utilization after medium-term residential assessment for epilepsy and psychogenic nonepileptic seizures. Epilepsy Behav. (2016) 62:147–52. 10.1016/j.yebeh.2016.06.004 27479776

[40] Ramamurthy S Steven Brown L Agostini M Lindstorm SA Dave H Dieppa M. Emergency department visits and readmissions in patients with psychogenic nonepileptic seizures (PNES) at a safety net hospital. Epilepsy Behav. (2021) 122:108225. 10.1016/j.yebeh.2021.108225 34352667

[41] Salinsky M Storzbach D Goy E Kellogg M Boudreau E. Health care utilization following diagnosis of psychogenic nonepileptic seizures. Epilepsy Behav. (2016) 60:107–11. 10.1016/j.yebeh.2016.04.007 27206227

[42] Asadi-Pooya AA. International multicenter studies on psychogenic nonepileptic seizures: a systematic review. Psychiatry Res. (2020). 10.1016/j.psychres.2020.112812 32014624

[43] Hingray C El-Hage W Duncan R Gigineishvelli D Kanemoto K LaFrance WC. Access to diagnostic and therapeutic facilities for psychogenic nonepileptic seizures: an international survey by the ILAE PNES Task Force. Epilepsia. (2018) 59:203–14. 10.1111/epi.13952 29152734

[44] FNDSociety. Functional Neurological Disorder. (2019). Available online at: www.fndsociety.org

[45] Edwards MJ Adams RA Brown H Pareés I Friston KJ. A Bayesian account of “hysteria.” *Brain*. (2012) 135:3495–512. 10.1093/brain/aws129 PMC3501967 22641838

