# Supplementary material for: Outcome of psychogenic non-epileptic seizures following diagnosis in the epilepsy monitoring unit
Source: Front Neurol. 2024 Feb 14;15:1363459. doi: 10.3389/fneur.2024.1363459 (PMC10899437; doi:10.3389/fneur.2024.1363459)
Supplement: Supplementary file 1 [file Data_Sheet_1.pdf]

Supplementary Figure 1: Participant selection process.

PNES = psychogenic non-epileptic seizure. In total, 360 patients admitted to our EMU from January 2009 to May 2023 were not diagnosed with epilepsy exclusively. This table illustrates the reasons which some patients were excluded. Ultimately, 61 patients were included in this study.

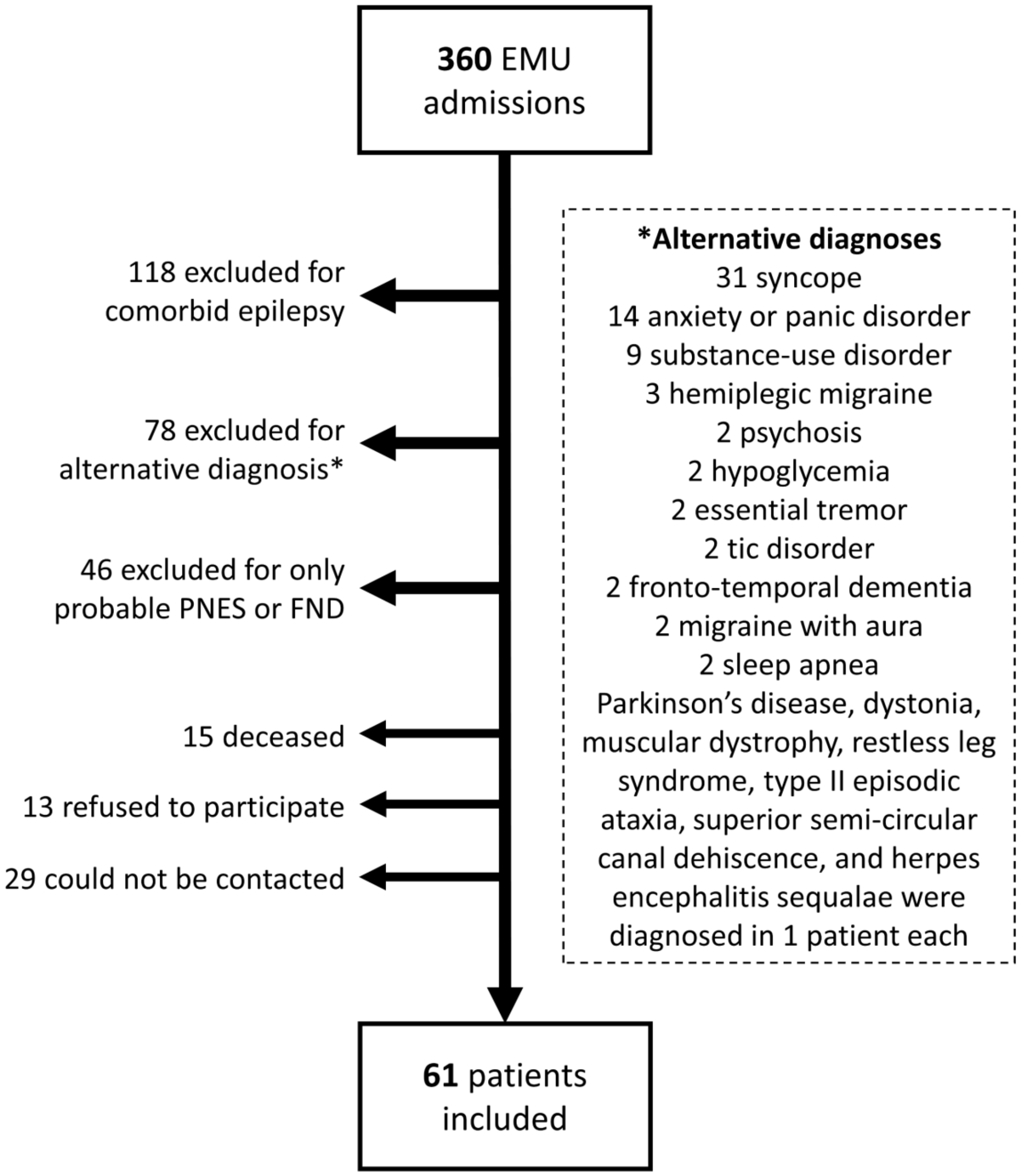

	n (%)	N
**Psychiatric comorbidities**	42 (69)	61
**Depressive Disorders:**	24 (39)	
Major Depressive Disorder	24 (39)	
**Anxiety Disorders:**	28 (46)	
Generalized Anxiety Disorder	22 (36)	
Panic Disorder	6 (10)	
**Neurodevelopmental Disorders:**	13 (22)	
ADHD	9 (15)	
Intellectual Developmental Disorder	2 (3)	
Autism Spectrum Disorder	1 (2)	
Tourette's Disorder	1 (2)	
**Personality Disorders:**	10 (16)	
Cluster B Personality Disorders:	8 (13)	
Borderline Personality Disorder	6 (10)	
Histrionic Personality Disorder	2 (3)	
Cluster C Personality Disorders:	3 (5)	
Dependent Personality Disorder	3(5)	
**Trauma- and Stressor-Related Disorders:**	7 (11)	
PTSD	7 (11)	
**Feeding and Eating Disorders:**	3 (5)	
Anorexia Nervosa	2 (3)	
Bulimia Nervosa	1 (2)	
**Bipolar and Related Disorders:**	2 (3)	
Bipolar disorder	2(3)	
**Obsessive-Compulsive and Related Disorders:**	2 (3)	
OCD	2 (3)	
**Schizophrenia Spectrum and Other Psychotic Disorders:**	1 (2)	
Delusional Disorder, Persecutory type	1 (2)	
**Dissociative Disorders:**	1 (2)	

Dissociative Identity Disorder	1 (2)	
**<u>Substance-Related and Addictive Disorders:</u>**	1 (2)	
Anxiolytic Use Disorder	1 (2)	
**Smoking**		61
Active	7 (11)	
Past	2 (3)	
Never	52 (85)	
**Alcohol**		61
Active (frequent)	3 (5)	
Active (occasional)	26 (43)	
Inactive/never	32 (52)	
**Cannabis**		61
Active (frequent)	7 (11)	
Active (occasional)	1 (2)	
Inactive/never	53 (87)	
**Other drugs**		61
Never	61 (100)	

Supplementary Table 2: Detailed psychiatric comorbidities and lifestyle habits of participants at PNES onset.

ADHD = attention-deficit/hyperactive disorder; n = count; N = number of complete cases; OCD = obsessive-compulsive disorder; PNES = psychogenic non-epileptic seizure; PTSD = post-traumatic stress disorder.

A single participant could have multiple psychiatric comorbidities.

	PNES freedom, n (%)
**Lifestyle modifications to prioritize mental health**	5 (18)
**Psychotherapy and better stress management**	6 (21)
**Understanding the diagnosis**	6 (21)
**No idea**	6 (21)
**ASM cessation**	4 (14)
**Antidepressants**	3 (11)
**Psychiatric care**	3 (11)
**Changing career path**	2 (7)
**Ending an abusive relationship**	0 (0)
**FND clinic**	1 (4)
**ASM use**	0 (0)
**Intrauterine device**	1 (4)
**CBD oil**	1 (4)
**Seeking legal action against aggressor**	1 (4)
**Stopping legal action against aggressor**	1 (4)
**Reading a book on PNES**	0 (0)

Supplementary Table 3: Factors reported by participants that led to their PNES freedom.

ASM = antiseizure medication; CBD = cannabidiol; FND = functional neurologic disorder; n = count; PNES = psychogenic non-epileptic seizure. One participant could name more than one factor. In total, there were 28 participants who were free of PNES.

**Employment status**	**At time of diagnosis, n (%)**	**After diagnosis, n (%)**
**Employed and working**	15 (25)	30 (49)
**On work leave**	28 (46)	16 (26)
**Never been employed**	3 (5)	3 (5)
**Student**	14 (22)	9 (15)
**Retired**	1 (2)	3 (5)

Supplementary Table 4: Employment status pre- and post-PNES-diagnosis.
